# Vegetables and Gout

**Published:** 1898-06-25

**Authors:** 


### VEGETABLES AND GOUT.

A paper read by Dr. Luff at the last meeting of the Royal Medical and Chirurgical Society contained the results of a series of researches made by the author as to the Influence on Gout of the Mineral Constituents of Various Vegetables. The outcome of the matter is, first, that as a matter of laboratory experience the salts contained in the ash of nearly all the vegetables experimented on very considerably increased the solubility of sodium biurate, the salt of which uratic deposits consist. But it is noteworthy that an artificially prepared ash of the same composition as the natural ash failed entirely to act in the same way, from which he opines that in the natural ash there is some combination of the mineral constituents which cannot be artificially imitated. Again, it was shown that the ash of some of the vegetables—notably of spinach, Brussels sprouts, French beans, cabbage, turnip tops, and turnips—very considerably delayed the conversion of the soluble quadriurate into the insoluble biurate, while the mineral constituents of meat diminished the solubility of the biurate and had but little effect in delaying the conversion of the quadriurate into the biurate. The result of these experiments was then to show that certain vegetables stood out prominently in regard to the influence on gouty changes exercised by their mineral constituents. These vegetables were spinach, Brussels sprouts, French beans, winter cabbage, Savoy cabbage, turnip tops, turnips, and celery; and he suggested that a table salt composed of the ashes of these vegetables might be used by the gouty in place of ordinary salt.

